# Supplementary material for: Oxylipins produced by Pseudomonas aeruginosa promote biofilm formation and virulence
Source: Nat Commun. 2016 Dec 8;7:13823. doi: 10.1038/ncomms13823 (PMC5155153; doi:10.1038/ncomms13823)
Supplement: Supplementary Information — Supplementary Figures 1-7. [file ncomms13823-s1.pdf]

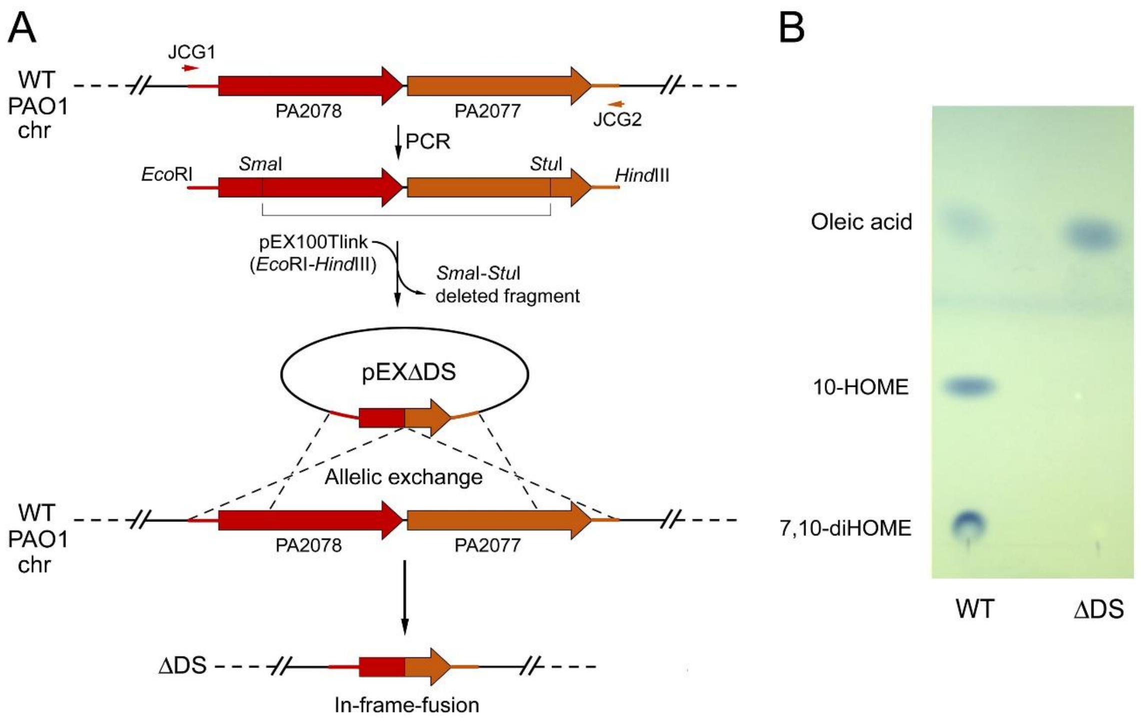

Supplementary Figure 1. Construction of a diol synthase mutant of *P. aeruginosa.* A) A fragment of *P. aeruginosa* chromosome containing the genes PA2078 and PA2077 encoding the diol synthase activity plus ~300 bp of chromosomal flanking regions were amplified by PCR using the primers JCG1 and JCG2. The fragment was cloned into the suicide vector pEX100Tlink and an internal *Sma*I-*Stu*I fragment was deleted to obtain pEXΔDS. This plasmid was used to make an in frame deletion of PA2077-78, through allelic exchange, in the chromosome of PAO1. The diol-synthase-activity-lacking strain obtained was named ΔDS. **B)** TLC plate showing that the ΔDS mutant lost the capacity to convert oleic acid into 10-HOME and 7,10-DiHOME oxylipins.

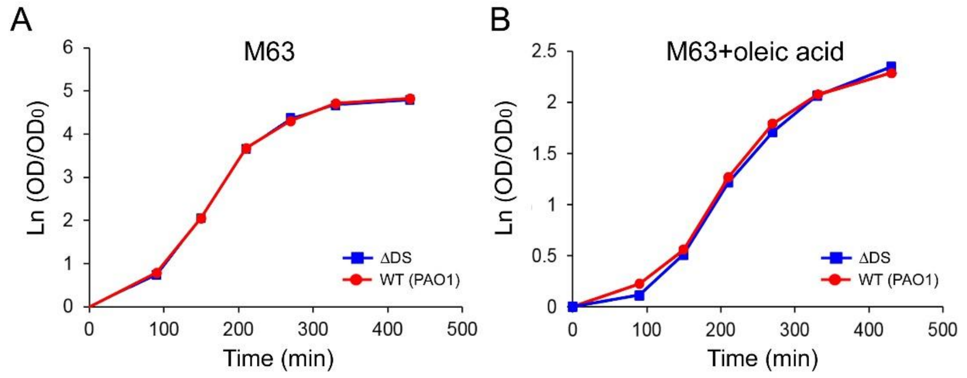

Supplementary Figure 2. The ΔDS mutant and its parental PAO1 display similar growth rates. **A)** ΔDS and PAO1 showed similar growth rates when they were grown in M63 medium, as well as when the M63 medium was supplemented with oleic acid at 0.1%. The slopes of the exponential phases of growth (calculated by linear regression) were similar. Values are averages from three independent experiments.

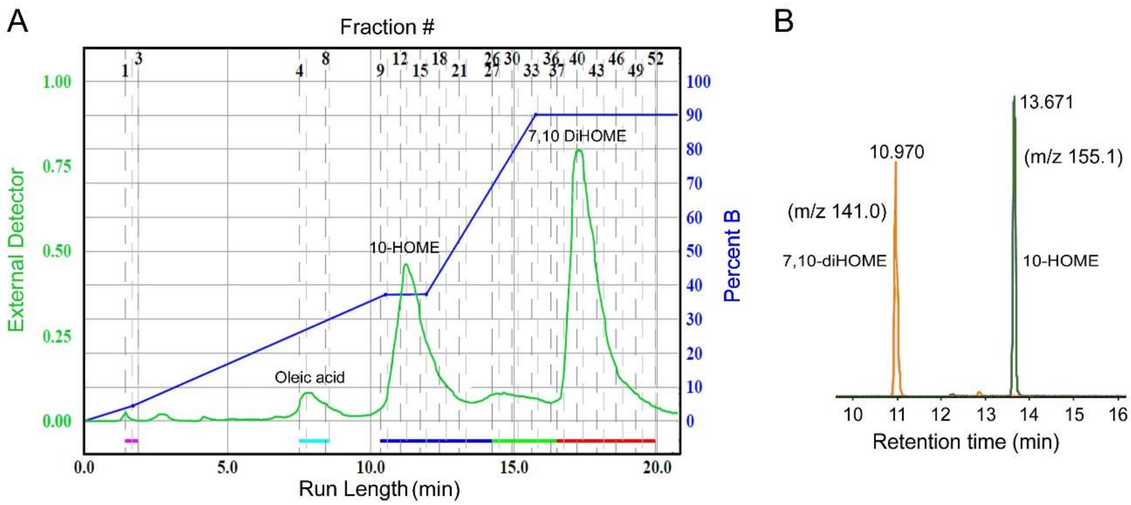

Supplementary Figure 3. Purification of 10-HOME and 7,10-DiHOME oxylipins from culture supernatants of *P. aeruginosa* PAO1. **A)** Graph showing the purification of 10-HOME and 7,10-DiHOME, which was carried out using a silica column and a gradient of ethyl acetate (solvent B) over hexane (solvent A). A good resolution of the peaks corresponding to 10-HOME and 7,10-DiHOME oxylipins was achieved applying the gradient curve shown in blue in the chromatogram. **B)** After the fractions for each detected peak were collected and combined, the purity and identity of the oxylipins was checked by HPLC/MS analysis. The reconstructed ion chromatograms of 10-HOME (*m/z* 155.1, in green) and 7,10-DiHOME (*m/z* 141, in yellow), which are shown superimposed in the graph, indicated that both oxylipins were obtained with a high grade of purity.

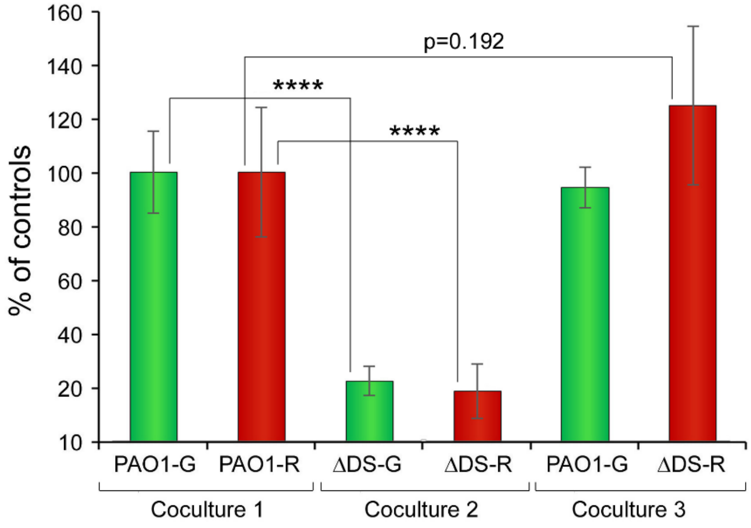

Supplementary Figure 4. The diol synthase mutant (ΔDS) behaves as a social cheater in co-culture with the wild type*.* When PAO1-derived strains expressing either GFP or RFP were co-cultured *(*PAO1-G, PAO1-R, coculture 1) the biofilms formed contained significantly more bacteria of each strain (t-test, ****, $p<0.0001$) than biofilms formed by coculture of ΔDS expressing either GFP or RFP (ΔDS-G, ΔDS-R, coculture 2) under the same conditions. However, when PAO1-G and ΔDS-R were cocultured (coculture 3), ΔDS-R incorporated into the biofilm to the same extent as PAO1 in coculture 1 (no significant difference, t-test, $p=0.192$), suggesting ΔDS cheats on oxylipins produced by PAO1. Results are expressed as percent of the amount of bacteria recovered from biofilms formed by cocultures of the WT strains PAO1-G and PAO1-R used as controls (coculture 1). Error bars represent SD of three independent experiments with at least four replicates each.

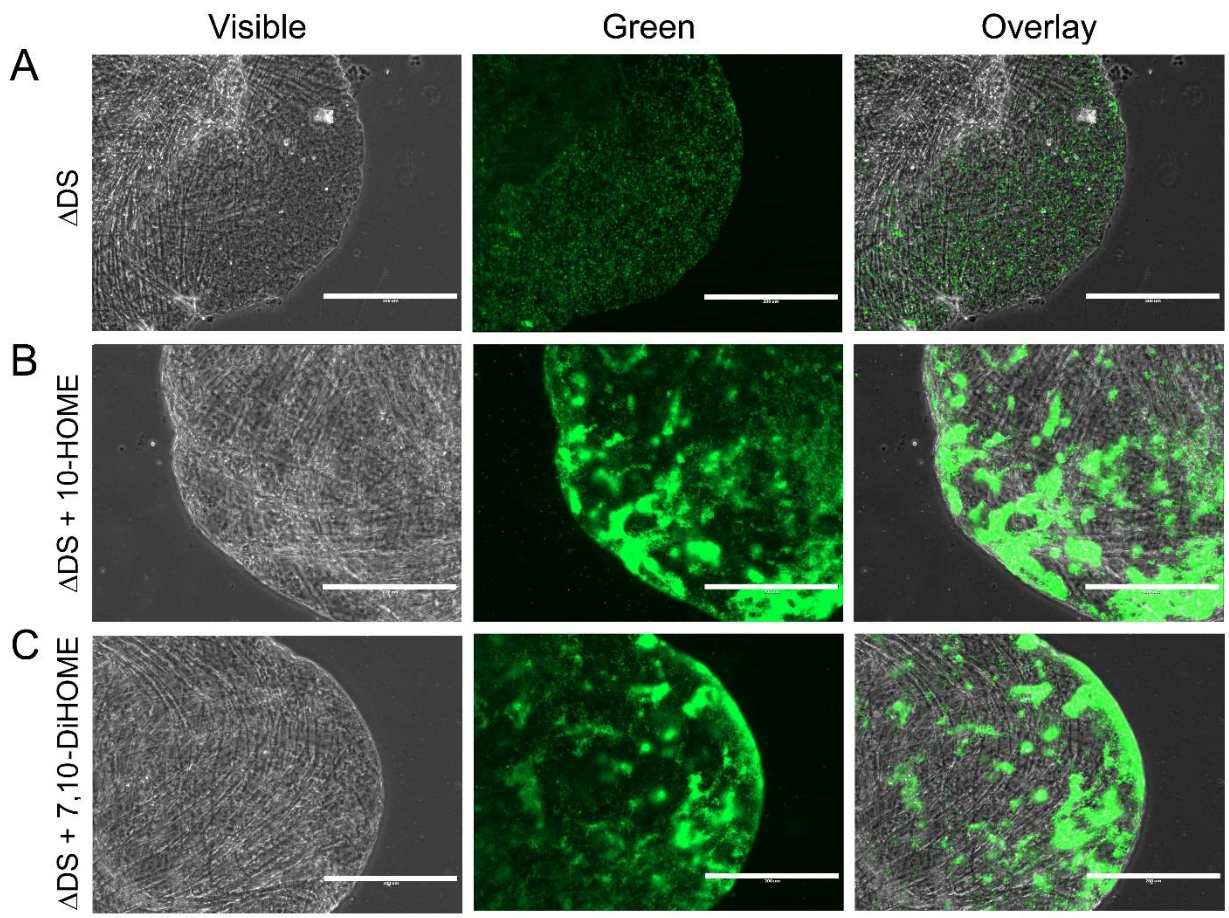

Supplementary Figure 5. Oxylipins promote biofilm formation of the ΔDS mutant in *D. melanogaster* crops. Fluorescence microscopy pictures of dissected crops from Drosophila flies fed with M63 media supplemented with 10-HOME or 7,10-DiHOME, into which GFP-expressing ΔDS strain was inoculated**.** A) As occurred with the WT PAO1 strain (Fig. 5), the ΔDS mutant formed very few microcolonies and the bacteria were mostly homogeneously distributed over the crop´s luminal epithelium. Also similar to PAO1, when the media was supplemented with 10-HOME (B) or 7,10-DiHOME (C) ΔDS was able to abundantly form microcolonies and early biofilm in flies' crops. Bars represent 200 µm. The size/resolution for each panel was adjusted to 2.125 x 1.587 in/600 dpi from 17.770 x 13.333 in/72 dpi of the originals. Pictures are representative of two independent experiments, in which five flies were dissected in each case.

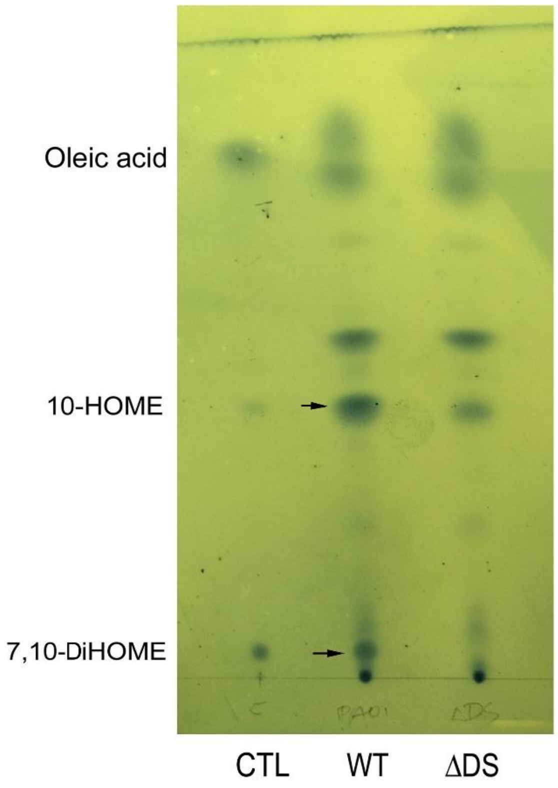

Supplementary Figure 6. *P. aeruginosa* produces 10-HOME and 7,10-DiHOME oxylipins in vivo. TLC image suggesting the presence of 10-HOME and 7,10-DiHOME in samples of fly homogenates inoculated with PAO1. Notice that spots with similar hydrophobicity to 10-HOME and 7,10-DiHOME showed in the control (left lane) are visible in the sample of flies inoculated with WT PAO1 (center lane) but not in ΔDS (right lane). The presence of the compounds was subsequently confirmed by HPLC/MS analysis (see Fig. 7C). As control we used a mix of oleic acid (Sigma O1008) plus purified 10-HOME and 7,10-DiHOME confirmed by MS.

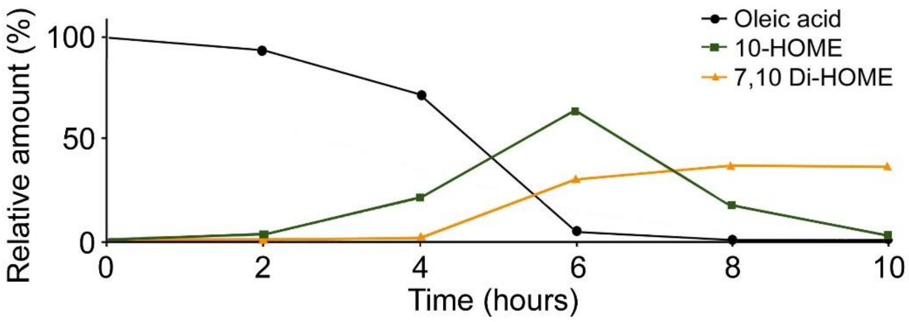

Supplementary Figure 7. Production kinetics of 10-HOME and 7,10-DiHOME oxylipins by *P. aeruginosa*. The kinetic study of production/consumption of 10-HOME and 7,10-DiHOME in *P. aeruginosa* culture supernatants, using oleic acid as a substrate at 1 mg/mL, shows that 10-HOME appears first in time while 7,10-DiHOME remains longer in the stationary phase of the culture. The graph plots relative semi-quantitative amounts of each compound calculated every 2 hours by densitometry of TLC spots using ImageJ software. Initial concentration of bacteria was adjusted at OD=1. Values are averages of two independent experiments.
